# Evaluating changeability to improve fruit and vegetable intake among school aged children

**DOI:** 10.1186/1475-2891-4-34

**Published:** 2005-11-21

**Authors:** Marilyn S Nanney, Debra Haire-Joshu, Michael Elliott, Kimberly Hessler, Ross C Brownson

**Affiliations:** 1 Department of Health Promotion & Education, University of Utah, Salt Lake City, UT; 2 Department of Community Health, School of Public Health, Saint Louis University, St. Louis, MO

## Abstract

**Background:**

The purposes of this paper are two fold. First, to describe an approach used to identify fruits and vegetables to target for a child focused dietary change intervention. Second, to evaluate the concept of fruit and vegetable changeability and feasibility of applying it in a community setting.

**Methods:**

Steps for identifying changeable fruits and vegetables include (1) identifying a dietary database (2) defining geographic and (3) personal demographics that characterize the food environment and (4) determining which fruits and vegetables are likely to improve during an intervention. The validity of these methods are evaluated for credibility using data collected from quasi-experimental, controlled design among 7–9 year old children (n = 304) participating in a tutoring or mentoring program in St. Louis, MO. Using a 28-item food frequency questionnaire, parents were asked to recall for their child how often foods were eaten the past 7 days. This questionnaire was repeated eight months later (response rate 84%). T-test analyses are used to determine mean serving differences from baseline to post test.

**Results:**

The mean serving differences from baseline to post test were significant for moderately eaten fruits (p < .001), however, not for vegetables (p = .312). Among the intervention group, significantly more children ate grapes (p < .001), peaches (p = .022), cantaloupe (p < .001), and spinach (p = .044) at post testing – all identified as changeable with information tailored to participants.

**Conclusion:**

Data driven, food focused interventions directed at a priority population are feasible and practical. An empirical evaluation of the assumptions associated with these methods supports this novel approach. However, results may indicate that these methods may be more relevant to fruits than vegetables. This process can be applied to diverse populations for many dietary outcomes. Intervention strategies that target only those changeable fruits and vegetables are innovative and warrant further study.

## Background

Children in the United States follow eating patterns that do not meet national recommendations for fruits and vegetables. [1,2] To date, dietary interventions have been only modestly successful in increasing fruit and vegetable (FV) intake among children [3] with effect sizes ranging from .2 servings (Gimme 5) [4] to .99 servings (High 5 Project). [5] One means of improving the impact of dietary interventions is to assure the intervention is relevant to a child by targeting foods that are available and accessible in their environment. [6]

Program planning models suggest that directing program resources toward those factors that are most *changeable *helps to ensure program efficiency and effectiveness. [7,8] For dietary interventions, the concept of changeability can be defined as identifying those FV that have the greatest likelihood for increased consumption and targeting them for an intervention. Changeability is moderated by variations in food consumption patterns across racial and ethnic, [9-12] gender, [9,10,12-15] age, [11,13,15] marital status, [11] and regional [11,13,16] differences. Changeability is also influenced by the discrepancies in the availability of food in the community and home environments, particularly in priority populations. [17-19]

From an individual perspective, the food experiences to which young children are exposed are critical to the early development of food acceptance patterns and choices. This exposure to FV influences familiarity, preferences, and intake. For purposes of an intervention, FV that are preferred and eaten by a child are less changeable because consumption goals are met. Instead, moderate intake of FV suggests foods that are familiar, accessible, and changeable. Systematic approaches derived from program planning constructs, are needed to determine program relevance and changeability in order to successfully impact health behaviors. [8]

The purpose of this paper is to describe a systematic approach used for identifying *changeable *FV that were targeted for intervention in Partners of all Ages Reading About Diet and Exercise (PARADE), a school based mentoring program designed to improve FV intake of children ages 7 to 9 years. Children enrolled in the mentoring program received one to one tutoring on a weekly basis during regular school hours. PARADE was incorporated within the routine curriculum of the mentoring program. PARADE was based on social cognitive theory and included (1) eight lesson plans with computer-generated storybooks tailored to the dietary patterns of each 7–9 year old child and (2) eight mailed parent newsletters introducing each book and offering tips on how to role model healthy eating at home. PARADE was evaluated using a quasi-experimental study design. Participants in the evaluation include children and their parents. Approval for this study was obtained from the Saint Louis University Institutional Review Board.

## Methods

### Steps for identifying *changeable *FV

Limited time and resources increased the importance of identifying appropriate foods for change in this priority population. The following steps were used to identify and tailor information on FV as part of PARADE lesson plans and storybooks.

#### Step 1: Identify a comprehensive nutrition database

Dietary measurement is complex. Accuracy can be maximized with methods that help individuals correctly recall the foods and amounts eaten on any given day that represents usual intake. Furthermore, a comprehensive list of foods and nutrient values is essential to calculating the dietary outcomes of interest. [20] The Continuing Survey of Food Intakes by Individuals (CSFII) database represents data from the priority population and is methodologically and psychometrically sound. Also popularly known as the "What We Eat in America Survey," the CSFII is a national food consumption survey conducted by the Agricultural Research Service of the United States Department of Agriculture. [21] The 10^th ^edition of the CSFII (1994–1996) provides nationally representative data by over sampling for low-income individuals and young children. Individuals are asked to provide food intakes on two nonconsecutive days using a multiple pass 24-hour dietary recall administered in the home by trained interviewers. Sample sizes include 12,700 adults of all ages and 11,800 children birth-19 years. Response rates include 1-day 80% and 2-day 76%. Food consumption data are available on CD-ROM from the USDA. More information, including ordering the CD's, can be found at .

#### Step 2: Define the sociodemographic characteristics of the priority population

Variations in dietary patterns are influenced by individual characteristics such as race, [9-12] gender, [9,10,12-15] and age. [11,13,15] Thus, it is critical to assess these factors with regard to determining changeability of fruits and vegetables. For the CSFII database, food consumption differences can be viewed by five race categories; white, black, Pacific Asian, Native American, and Other. Furthermore, the data can be segmented by gender and age. The CSFII database reports age in months for children less than one year of age, and in years for those over 1 year of age. For Project PARADE, the CSFII data was analyzed for children 7–9 years of age (9% of CSFII sample), male (49%) and female (51%), and African American (18%). 892 children matched these criteria.

#### Step 3: Define the geographic characteristics of the priority population

Dietary intake varies by geographic region and urban versus rural influences. The CSFII divides the United States into five geographic regions that are further defined by urbanization type. Urbanization types include city, outside the city, and rural areas. PARADE participants lived in ten urban and suburban counties in a large Midwest city. Therefore, the CSFII data was analyzed for those living in a metropolitan city and outside the city in the Midwest. Results identified nearly three-quarters of the sample living in a metropolitan area (central city, 21% and suburban, 53%). These geographic criteria resulted in a final sample of dietary intake data for 164 children, mean age 7.93 (SD = .82). Steps 1–3 identified 2423 food entries by the CSFII. This included 670 unique foods including 49 fruits and 79 vegetables consumed by the sample.

#### Step 4: Identify changeable FV for the priority population

Individual characteristics and environmental exposures influence food familiarity and determine frequency of consumption. We next selected those foods meeting criteria for 'moderate consumption'. CSFII food consumption data is organized by numerical food codes and food amounts. Data can be viewed in a number of ways; specific food, food form (canned, frozen, raw), how often a food was eaten, and the amount eaten (gram weight). Individual food items were rank ordered by the percentage of respondents who reported consuming them over the observation period. Foods whose rank fell into the 25^th ^to 75^th ^percentile were defined as "moderately consumed". Five fruits and eight vegetables were identified as moderately consumed and targeted for an intervention among these school-aged children. Foods in the top quartile (>75%) or most frequently consumed (i.e., oranges, carrots) and FV at the lower range (<25%) of consumption (i.e., grapefruit, sweet potatoes) were not included in the intervention. Table 1 summarizes the steps that define the key characteristics to consider when determining relevant foods for a dietary intervention.

**Table 1:** Steps to identify relevant foods to target in a dietary intervention

**Steps to Determine Foods for a Dietary Intervention**	**Key Characteristics that Effect Dietary Intake**
Define Sociodemographic Variables	**Marital status** • Married, Divorced, Separated, Single, Widowed
	**Race** • Caucasian, African American, Pacific Asian, Native American, and Other
	**Gender** • Male, Female
	**Age** • 0–12 Months, Years
Define Geographic Variables	**Regional** • North, South, East, West, Midwest
	**Urbanization** • City, Suburban, Rural
Identify Changeable Foods to Target	**Consumption Frequency** • Rank % between 25 and 75

## Results

### Using national frequency data to estimate local FV consumption

An important assumption being made is that accurate conclusions can be drawn from food consumption frequencies collected from a national sample because they mirror local consumption patterns. Table 2 compares FV consumption frequency identified from the CSFII with baseline PARADE data (intervention and delayed intervention groups) and indicates agreement between CSFII rank % and PARADE rank % consumptions. For example, according to the national data, those FV eaten most frequently (oranges, juice, apples, potatoes, lettuce) correspond with the PARADE baseline data as indicated by the 76–100 rank percents. Similar agreement occurs for those fruits and vegetables eaten moderately (25–75 rank percent) and least often (<25 rank percent). However, a few discrepancies are noted. Although not identified as a moderately eaten fruit from the national data set, kiwi falls within the moderately eaten range at PARADE baseline (13% versus 31%) and therefore should have been targeted as changeable in the intervention. Vegetable consumption was less congruent than fruit consumption. Two vegetables (corn, carrots) were eaten more often in the national sample than reported by PARADE children at baseline. Conversely, PARADE children ate green beans and cabbage slaw more often. Overall, the authors conclude that using national dietary data to identify percent rank cut points defining consumption frequency can be useful when developing community interventions. These methods may be more accurate in predicting fruit consumption than vegetable consumption.

**Table 2:** Fruits and vegetables identified as changeable and targeted for intervention

**Fruit**	CSFII Weighted % Consumed^1 ^(n = 502)	CSFII Rank %	PARADE Baseline % Consumed^2 ^(n = 304)	PARADE Baseline Rank %
Oranges/juice	24.3%	100%	79.6%	100%
Other juice	17.3%	94%	67.8%	94%
Apples	13.4%	88%	49.7%	88%
Bananas	9.0%	81%	49.0%	81%
^CF ^Fruit Cocktail	4.5%	**75%**	32.9%	**56%**
^CF ^Grapes	4.2%	**69%**	46.4%	**75%**
^CF ^Peaches	2.8%	**50%**	28.9%	**25%**
^CF ^Cantaloupe	2.4%	**44%**	17.4%	**31%**
^CF ^Strawberries	2.1%	**31%**	43.7%	**63%**
^CF ^Pineapple	1.8%	**25%**	20.1%	**38%**
Kiwi	.84%	13%	18.2%	***31%***
Nectarines	.48%	6%	15.2%	19%
Grapefruit	.12%	0%	9.2%	6%

**Vegetables**	CSFII Weighted % Consumed^1^	CSFII Rank %	PARADE Baseline % Consumed^2^	PARADE Baseline Rank %

Potatoes	48.4%	100%	95.1%	100%
Lettuce	12.1%	89%	62.2%	79%
Corn	9.6%	84%	52.6%	***63%***
Carrots	8.7%	79%	54.6%	***68%***
^CV ^Green beans	7.62%	**74%**	72.0%	*89%*
^CV ^Mixed Vegetables	6.3%	**68%**	46.7%	**53%**
^CV ^Tomatoes	5.2%	**63%**	49.0%	**58%**
^CV ^Cabbage slaw	2.8%	**47%**	13.2%	*11%*
^CV ^Beans	4.3%	**53%**	25.3%	**26%**
^CV ^Green Peas	2.7%	**42%**	38.2%	**37%**
^CV ^Broccoli	2.4%	**32%**	55.3%	**74%**
^CV ^Spinach/greens	1.2%	**26%**	52.6%	**63%**
Bell Pepper	1.1%	16%	12.1%	5%
Sweet potatoes	.6%	11%	14.2%	16%
Cauliflower	.5%	5%	18.2%	21%

^CF ^= Changeable Fruits

^CV ^= Changeable Vegetables

^1 ^CSFII weighted percent consumed was calculated from the percentage of respondents who reported consuming each food item at least once during the period under observation (two days). These data were weighted to represent urban and sub-urban Midwesterners using weights provided by the CSFII study.

^2 ^PARADE baseline percent consumed was calculated from the percentage of respondents who reported consuming each food item during the past week.

After identifying FV for intervention, we incorporated this information into 8 tailored storybooks. Professionally trained interviewers then administered a telephone survey to parents of children enrolled in participating tutoring programs. Using a 28-item FV food frequency questionnaire, parents were asked to recall for their child how often select foods were eaten within the past 7 days. This questionnaire was repeated eight months later. Response choices included "none, 1 time, 2 times, 3–4 times, 5–6 times, 7 or more times in the past week". The questionnaire demonstrated acceptable internal consistency (Chronbach's alpha = .744). Table 3 presents the PARADE changeability results. Six PARADE fruits (PF) and eight vegetables (PV) identified as moderately consumed are hypothesized to be the most likely to improve from baseline to post intervention. Differences in the percent of children who consumed the "same (unchanged) or more" of each PF and PV from baseline to posttest indicate that those targeted as changeable in the intervention were more likely to be eaten.

**Table 3:** Fruits and vegetables targeted for Project PARADE improved at post intervention

**Fruit**	**Pre-Post Change**	**p-value**	**Average Pre % consumed**	**Average Post % consumed**
Oranges/Juice	Unchanged	.464		
Other Juice	Unchanged	.138	62%	61%
Apples	Unchanged	.139		
Bananas	Unchanged	.414		
^PF ^Fruit Cocktail	Unchanged	.387		
^PF ^Grapes	**More**	**<.001**		
^PF ^Peaches	**More**	**.022**	32%	38%
^PF ^Cantaloupe	**More**	**<.001**		
^PF ^Strawberries	Unchanged	.355		
^PF ^Pineapple	Unchanged	.426		
Grapefruit	Unchanged	.149	14%	7%

**Vegetable**	**Pre-Post Change**	**p-value**	**Average Pre % consumed**	**Average Post % consumed**

Potatoes	**More**	**.025**		
Lettuce	Unchanged	.118	66%	71%
Corn	**More**	**.044**		
Carrots	Unchanged	.268		
^PV ^Green beans	Unchanged	.456		
^PV ^Mixed Vegetables	Unchanged	.062		
^PV ^Tomatoes	Unchanged	.230		
^PV ^Cabbage slaw	Unchanged	.500	44%	45%
^PV ^Beans	Unchanged	.202		
^PV ^Green Peas	Unchanged	.440		
^PV ^Broccoli	Unchanged	.441		
^PV ^Spinach/greens	**More**	**.044**		
Sweet potatoes	Unchanged	.500	15%	14%
Other vegetables	Unchanged	.470		

^PF ^= PARADE Fruits

^PV ^= PARADE Vegetables

Among the intervention group, significantly more children ate grapes (p < .001), peaches (p = .022), cantaloupe (p < .001), and spinach (p = .044) at post testing – all identified as changeable with information tailored to PARADE participants. As hypothesized, consumption of fruits and vegetables frequently and rarely eaten remained unchanged or were eaten more in two instances (potatoes, corn). Worth noting is that the majority of the vegetables remained unchanged at the end of the intervention. These results may indicate that these methods may be more relevant to fruits than vegetables.

Table 4 summarizes the results by grouping all frequently, moderately, and rarely consumed FV. Average (or mean) baseline servings among PARADE intervention participants (N = 304) are consistent with the assumptions presented where those described as frequently (Mean = 1.75 servings), moderately (Mean = 0.75 servings), and rarely (Mean = 0.26 servings) consumed follow that pattern. T-test analyses indicate that mean serving differences from baseline to post test were significant for moderately eaten fruits (p < .001), however, not for vegetables (p = .312). In general, the authors conclude that applying the concept of changeability to a FV intervention among school-aged children is feasible and supported by this data.

**Table 4:** Moderately eaten fruits improved at post intervention

PARADE Intervention (N = 304)				
	Mean Servings Baseline	Mean Servings Post	T-test Difference	p-value

Frequently Eaten Fruits	1.75	1.72	.504	.615
**Moderately Eaten Fruits**	**.746**	**.932**	**-4.15**	**<.001**
Rarely Eaten Fruits	.264	.213	.802	.423
Frequently Eaten Vegetables	1.36	1.41	-1.43	.155
Moderately Eaten Vegetables	.720	.745	-1.013	.312
Rarely Eaten Vegetables	.181	.159	.393	.695

## Discussion

Recent findings suggest that innovative research is necessary to broaden the traditional approach beyond increasing FV awareness and education. [22] Overall, this study reinforces these methods as a way to systematically identify FV to target for an intervention. Identifying and concentrating on the most changeable behavioral targets for interventions direct resources to where they will be most beneficial. Furthermore, greater specificity in program development simplifies the evaluation process (i.e., brief food frequency questionnaire). [23,24]

Assessments of changeability across fruit and vegetable patterns can be made by defining person and place variables of the priority population, examining the frequency with which fruits and vegetables have been consumed, and identifying those that can be reasonably expected to change for targets of an intervention. Moreover, this approach takes into account important environmental influences upon dietary patterns. Haire-Joshu and Nanney describe individual food preference, cultural and familial influences, and home, school and community environments as having significant influences upon the food environment of children. [6] This process addresses eating behavior as a function of the varied food environments for a specific population, albeit, in a broader community based context.

We developed, applied, and tested a systematic, data based approach to assess changeability and specify fruits and vegetables for an intervention among underserved school aged children. This approach will allow for further clarity of intervention effects by targeting only those changeable fruits and vegetables for intervention. Furthermore, this process can be applied to diverse populations for a variety of dietary outcomes. Additionally, larger mass media interventions like the 5 A Day campaign may benefit from this approach. More research is needed to evaluate the effectiveness and generalizability of community-based efforts that promote changeable foods for an intervention.

## Competing interests

The author(s) declare that they have no competing interests.

## Authors' contributions

MSN conceived of the concept of changeability and applied the methods to the intervention, developed the data collection instrument and drafted the manuscripts. DHJ conceived of the study, and participated in its design and coordination and helped to draft the manuscript. ME managed the data, performed statistical analysis, and helped to draft the manuscript. KH contributed to the conception of the study, application of the methods, and helped draft the manuscript. RB participated in the design of the study, oversaw the statistical analysis, and helped to draft the manuscript.
